# A novel antibody surrogate biomarker to monitor parasite persistence in *Trypanosoma cruzi*-infected patients

**DOI:** 10.1371/journal.pntd.0006226

**Published:** 2018-02-09

**Authors:** Maan Zrein, Elodie Granjon, Lucie Gueyffier, Julie Caillaudeau, Peter Liehl, Hans Pottel, Clareci Silva Cardoso, Claudia Di Lorenzo Oliveira, Lea Campos de Oliveira, Tzong-Hae Lee, Ariela Mota Ferreira, Antonio Luiz P. Ribeiro, Michael P. Busch, Ester Cerdeira Sabino

**Affiliations:** 1 R&D, INFYNITY BIOMARKERS SAS, Lyon, France; 2 Faculty of Medicine, University of Leuven (KULAK), Kortrijk, Belgium; 3 Federal University of São João del-Rey, Research Group in Epidemiology and New Technologies in Health-campus CCO, São João del-Rei, Brazil; 4 Instituto de Medicina Tropical e Faculdade de Medicina (FMUSP) da Universidade de São Paulo, São Paulo, Brazil; 5 Laboratorio de Investigação Médica (LIM03), Hospital das Clinicas da Faculdade de Medicina da Universidade de São Paulo, São Paolo, Brazil; 6 Blood Systems Research Institute and University of California, San Francisco, California, United States of America; 7 Graduate Program in Health Sciences, State University of Montes Claros (Universidade Estadual de Montes Claros), Montes Claros, Minas Gerais, Brazil; 8 Hospital das Clínicas and School of Medicine, Universidade Federal de Minas Gerais, Belo Horizonte, Brazil; Universidade Federal de Minas Gerais, BRAZIL

## Abstract

**Background:**

*Trypanosoma cruzi* parasite, the causative agent of Chagas disease, infects about six million individuals in more than 20 countries. Monitoring parasite persistence in infected individuals is of utmost importance to develop and evaluate treatments to control the disease. Routine screening for infected human individuals is achieved by serological assays; PCR testing to monitor spontaneous or therapy-induced parasitological cure has limitations due to the low and fluctuating parasitic load in circulating blood. The aim of the present study is to evaluate a newly developed antibody profiling assay as an indirect method to assess parasite persistence based on waning of antibodies following spontaneous or therapy-induced clearance of the infection.

**Methodology/Principal findings:**

We designed a multiplex serology assay, an array of fifteen optimized *T*. *cruzi* antigens, to evaluate antibody diversity in 1654 serum samples from chronic Chagas patients. One specific antibody response (antibody 3, Ab3) showed a strong correlation with *T*. *cruzi* parasite persistence as determined by *T*. *cruzi* PCR positive results. High and sustained Ab3 signal was strongly associated with PCR positivity in untreated patients, whereas significant decline in Ab3 signals was observed in BZN-treated patients who cleared parasitemia based on blood PCR results.

**Conclusion/Significance:**

Ab3 is a new surrogate biomarker that strongly correlates with parasite persistence in chronic and benznidazole-treated Chagas patients. We hypothesize that Ab3 is induced and maintained by incessant stimulation of the immune system by tissue-based and shed parasites that are not consistently detectable by blood based PCR techniques. Hence, a simple immunoassay measurement of Ab3 could be beneficial for monitoring the infectious status of seropositive patients.

## Author summary

Chagas disease is a zoonosis caused by *Trypanosoma cruzi* infection. In this study, we sought to identify a simple serologic biomarker that can supersede PCR techniques in characterizing patients for parasite persistence, whether circulating or shielding in tissues. A multiparametric assay was designed to evaluate antibody diversity in untreated and benznidazole-treated chronic cardiomyopathy patients. Out of 15 different antigens we identified a distinct antibody specificity that strongly correlates with parasite persistence. This putative biomarker could be used to monitor active infection in *T*. *cruzi* seropositive patients and identify patients that responded to anti-parasitic therapy.

## Introduction

Chagas disease, also named American Trypanosomiasis, is considered one of the 17 neglected illnesses by the World Health Organization [1]. It affects over 6 million persons worldwide, mainly in Latin America where the disease is endemic [2]. Chagas disease is caused by *Trypanosoma cruzi (T*. *cruzi)*, a protozoan parasite disseminated by a Triatome vector. Such insects have a large reservoir territory that spans from Argentina/Chile to the southern part of the United States, raising concerns on a possible spreading of the disease outside Latin America. Moreover exposed population are migrating to other continents, inducing the emergence of cases in non-endemic regions [3]. The disease may also be transmitted through blood transfusion, congenitally or by oral route thru consumption of insect-infested fruit-products.

Different clinical forms of Chagas disease exist. Patients in the acute phase of infection are generally symptom-free or have mild non-specific symptoms [1]. Following this acute infection period (weeks or months) and in the absence of an adequate treatment, the infection generally progresses from acute to chronic phases, with a small proportion of spontaneously resolved infections [4]. The majority of chronic Chagas disease (CC) patients remain in “indeterminate form”, defined as asymptomatic persistent infection that can last for decades. However, about 30% of patients eventually develop life-threatening cardiac and/or digestive symptoms requiring complex clinical management and specific treatments [5].

Two drugs are currently available to treat acute or CC infection: benznidazole (BZN) and Nifurtimox introduced in 1971 and 1965, respectively [6]. These treatments show different levels of efficacy that are difficult to evaluate, in particular during the chronic/indeterminate phase. Currently the main criterion to establish treatment success (parasitological cure) is the reversion of the conventional serology from positive to negative status in association with negative results on parasitological tests (xenodiagnosis and/or heamoculture, or PCR-based parasite assays) [7] [8]. During the acute phase, both drugs have shown successful clinical, parasitological, and serological treatment outcomes. Similarly, congenital Chagas disease was also successfully cured [9]. However, during the late CC phase (infections for more than ten years) [10], the serological cure rate with BZN is estimated to be between 8% and 40% [11] [12]. Thus, treatment efficacy for adult patients in the chronic phase is still controversial and difficult to estimate. For example, some studies have shown that treatment reduced the progression rate to cardiomyopathy [13], whereas the large but controversial BENEFIT study indicated that the drug did not improve cardiac outcomes when given to patients with chronic cardiomyopathy [14] [15]. Nonetheless, it is generally agreed that BZN-treatment contributes to decreasing the parasitic load resulting in a decline in antibody titers, and consequently is likely to improve clinical outcomes if applied early in the chronic phase of the infection [14] [16] [17]. Several other factors also affect the drug success rate such as the genotype of the parasite, the age of the patients, the interval between infection and start of treatment, and the clinical stage of the disease when therapy is given [11] [18].

In addition to being only moderately successful, the treatment causes many side effects, with dermatitis the most frequently observed. Other serious side effects are also reported, such as digestive manifestations, hypersensitivity reactions and polyneuropathy [19]. For these reasons, the risk-benefit ratio between adverse events and expected benefits is taken into account for patients with or at risk for cardiac and/or digestive complications [9].

In the absence of specific clinical signs, several techniques have been proposed to evaluate the treatment efficacy of Chagas disease including serological assays and detection of the parasite in blood by PCR. Among these techniques, PCR has shown promising results for assessment of anti-parasitic therapy failure, but not therapy efficacy [20]. Indeed a positive parasitological result after treatment is considered unequivocal evidence of failure to clear the parasite and thus ineffective treatment [21]. However, PCR negative results do not ensure the absence of infection given that the parasitemia fluctuates between detectable and undetectable concentrations in blood [21] and the parasite may persist in host tissues like muscle or may intermittently circulate at a very low or undetectable levels in blood [22]. Another technique that has been evaluated for the follow-up of treatment efficacy is serology testing. Several reports have shown that a reduction of antibody levels can be used to monitor the early impact of treatment [11] [21] [23]; such reduction can ultimately attain full “sero-reversion” from positive to negative status on sensitive screening assays, but this may require up to twenty years depending on the sensitivity of the antibody assay. Finally, there is no recognized gold standard cure marker [24] and the identification of early biomarkers of persistence or clearance of the parasite is needed, not only in symptomatic patients but also in the indeterminate forms of the disease. Such biomarkers are of utmost importance to improve patient management and evaluation of efficacy of new drugs under development [25] given the low efficacy and toxicity of the existing drugs.

The aim of the study we present in this manuscript was to characterize serological profiles of *T*. *cruzi* -infected patients on an extended version of the MultiCruzi assay [26]. By measuring diversity of antibodies we sought to identify surrogate antibody biomarker(s) that could be sensitive and specific for monitoring parasite persistence regardless of PCR results. For this purpose, we evaluated a large sample collection from the SaMi-Trop cohort [27] including chronic Chagas cardiomyopathy patients treated with BZN and an untreated control group, all tested by PCR (n = 1654).

## Materials and methods

### Ethics statement

All human subjects were adult. The experimental plan of the research study was in agreement with the Declaration of Helsinki and was approved by a local institutional ethical committee for each sampling site (Conep) and by the University of Sao Paulo Medical IRB (ref. 00580612.8.0000.0065). Written informed consent was obtained for patients after a detailed explanation on the usage for research purpose of their blood samples.

### Study population and samples origin

The SaMi-Trop project is a prospective cohort study of 1959 patients with chronic Chagas cardiomyopathy conducted in 21 cities of the northern part of Minas Gerais State in Brazil (S1 Appendix) [27]. The aim of this cohort establishment was to find biomarkers associated with the risk of death or evolution towards more severe disease. The inclusion criteria were individuals with ECG abnormalities and tested positive for the serology of Chagas disease. The exclusion criteria included pregnancy or breast feeding, and any life-threatening disease with an ominous prognosis. The patients included in the cohort aged between 28 to 91 years with an average age of 57 years. When first enrolled in the study, two PCR assays were performed on large blood samples from each patient and the patients were interviewed using a standardized questionnaire. This questionnaire provided information including history of previous treatment for Chagas disease and the duration since of the first reported exposition to *T*. *cruzi* [27].

Of the initial 1959 available serum samples, 135 were excluded because the information regarding history of previous treatment was missing, and 170 showed technically invalid results that require repeated testing. Hence, among the SaMi-Trop patients, we tested 1654 fully documented samples from patients without self-reported previous treatment (n = 1199) or with a history of BZN treatment (n = 455) for antibody profiles. Among these treated patients, the reported treatment history ranged from one year to ten years prior to accrual.

### Multiplex Chagas assay

**Fig 1:**
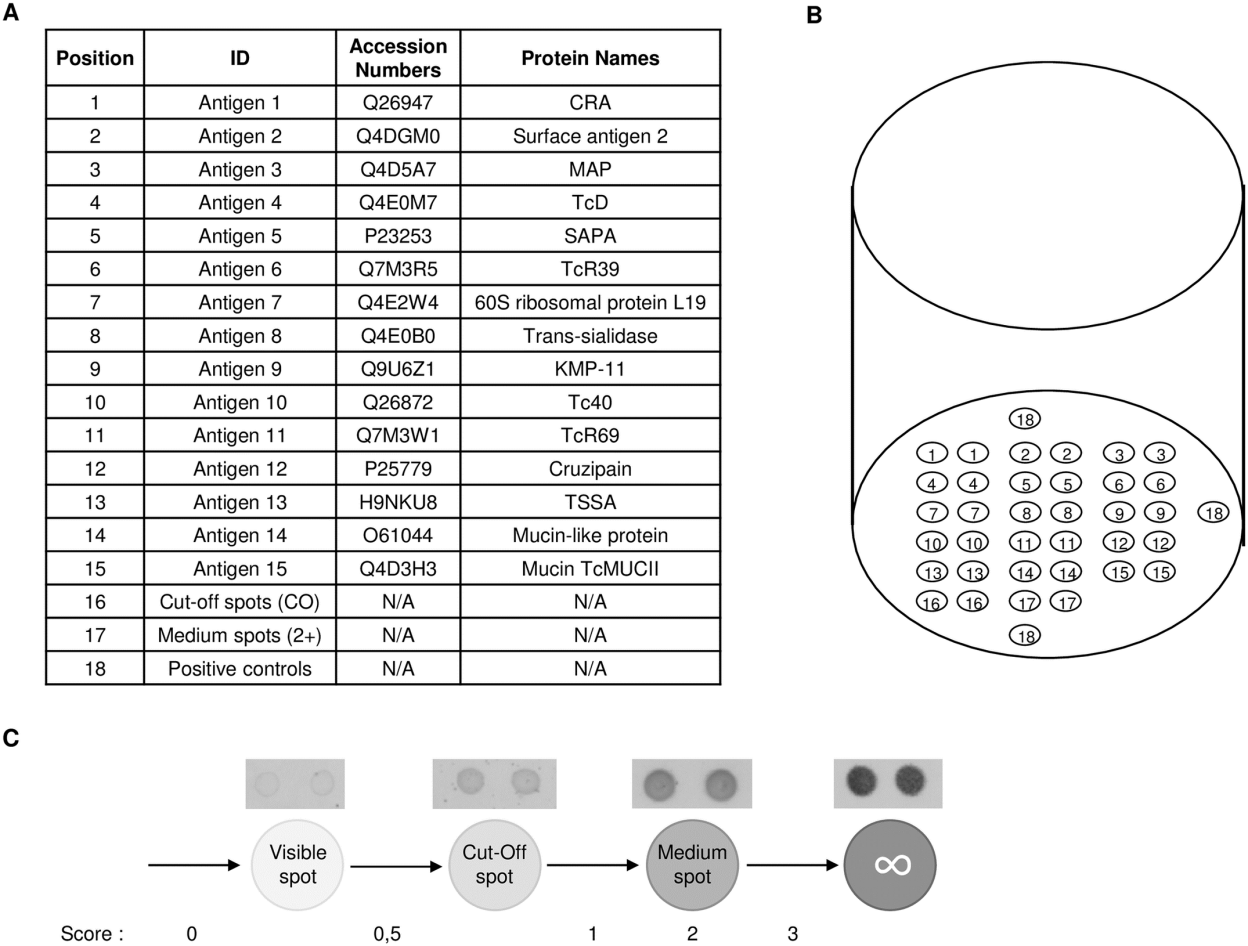
Antigens map. (A) Antigens used in the assay and their respective position in the array are given together with the corresponding references. (B) Schematic representation of the fifteen antigens spotted in duplicate in a single well of a 96-well plate. (C) For the visual interpretation, the intensity of each antigen is compared to the range spots (cut-off and medium spot) and the intensity is scored as follows: 0, absent or less intense than a visible spot; 0.5, intensity between that of the visible spot and that of the cut-off spot; 1, intensity between the cut-off spot and that of the medium spot; 2, intensity equal to that of the medium spot; 3, intensity higher than that of the medium spot.

The assay presented in this study is an extended version of the MultiCruzi confirmatory assay previously described [26]. We used the sciFLEXARRAYER system (SCIENION, Germany) to print fifteen *T*. *cruzi* antigens (Fig 1A), selected for their proven immunogenic properties, in duplicates in each well of a 96-well plate. Relevant antigens are designed then obtained synthetically according to the reviewed and published non-redundant sequences available at the repository “www.uniprot.org”. The list of accession numbers is detailed on Fig 1A next to each antigen position (1 to 15). Among the fifteen antigens, three antigens were strain-specific derived from the sequences of TcI, TcII and TcVI strains of *T*. *cruzi*. In addition to these antigens, the positive control spots were printed in triplicates according to a precise spatial orientation pattern (Fig 1B). These embedded controls were designed to check the sequential addition of all reagents (human serum samples, enzyme conjugates and substrate). The coating of antigens and the ELISA assay were carried out as previously described [26]. In order to obtain a visual interpretation at the end of the test, we also embedded cut-off control and medium control spots. By comparing intensity of each antigen to both spots, one can assign a score between 0 and 3 to each of the fifteen measured antibodies (named “reduced-scoring”; see Fig 1C). More generally, the assay-result images could be analyzed in two ways, visually or using a digital microplate reader. For the visual interpretation, each antigen was compared to the scoring spots (cut-off and medium controls) and the intensity was scored as follows: 0, absent or less intense than a visible spot; 0.5, intensity between that of the visible spot and that of the cut-off spot; 1, intensity between the cut-off spot and that of the medium spot; 2, intensity equal to that of the medium spot; 3, intensity higher than that of the medium spot (Fig 1C). When using a digital reader, we obtained a net intensity (mean value of duplicated spots intensity) for each of the fifteen measured antibodies. This net intensity can be converted to a reduced scoring information using the same approach as visual reading detailed above.

### PCR analysis

The target-capture real-time (RT) PCR assay used in this study was developed based on the PCR method that targets satellite *T*. *cruzi* DNA [28]. DNA extraction and concentration was improved through use of a target capture step that employed magnetic beads coated with *T*. *cruzi*-specific 20-mer capture oligonucleotides:

Capture TCZ 1 CGAGCTCTTGCCCACACGGGAAAAAAAAAAAAAAAAAAAAAAAAAA;
Capture TCZ 2 CCTCCAAGCAGCGGATAGTTCAGGAAAAAAAAAAAAAAAAAAAAAAAAAA;
Capture TCZ 3 TGCTGCASTCGGCTGATCGTTTTCGAAAAAAAAAAAAAAAAAAAAAAAAAAA;
Capture TCZ 4 CARGSTTGTTTGGTGTCCAGTGTGTGAAAAAAAAAAAAAAAAAAAAAAAAAAA.

Two replicate assays were performed and results interpreted as positive if the two replicates were positive. If a single positive was observed, the samples were repeated with four replicates and results were considered positive if at least two of four replicates were positive. A cycle threshold (CT) of 45 was the cut-off CT. Quantification of parasitemia was based on a curve obtained from parasites culture, with 0.1 to 10,000 parasites/mL [28].

### Statistical analysis

**Fig 2:**
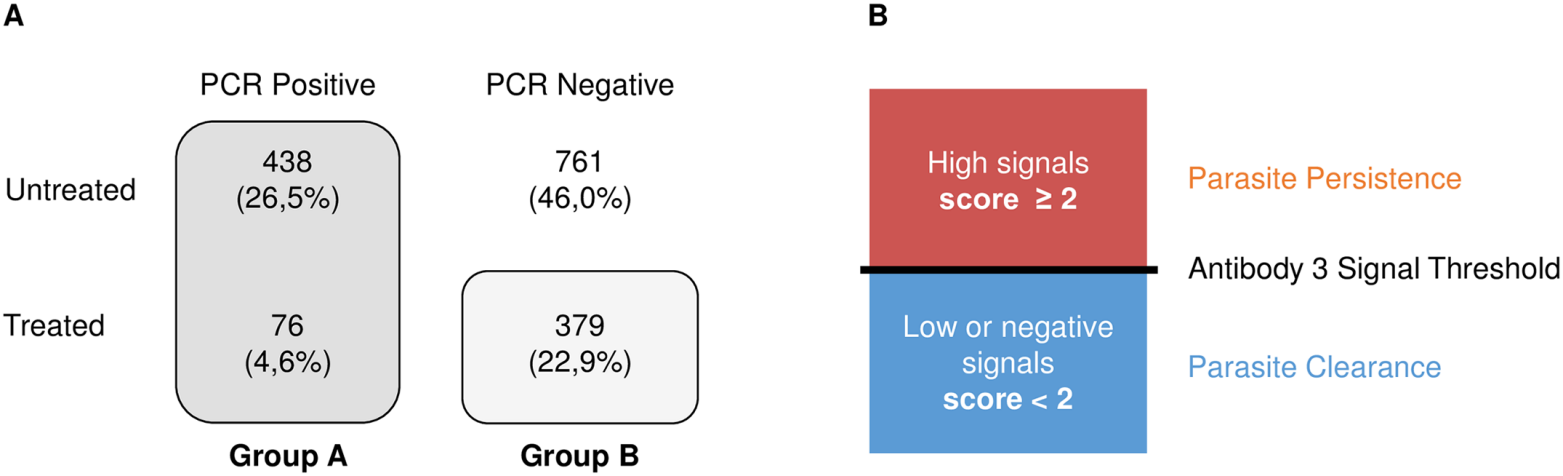
Identification of Ab3 biomarker. (A) Scheme illustrating the two groups used to identify antibody biomarker(s) that could serve as parasite persistence indicator. Group A comprises untreated and treated PCR Pos patients because all of them contain circulating parasites. Group B includes treated PCR Neg patients because they have the highest likelihood of parasitological cure. (B) Algorithm for defining parasite persistence versus parasite clearance based on Ab3. High signal intensity of Ag3 are assessed with a score ≥ 2 indicating parasite persistence; low or negative Ag3 are evaluated with a score < 2 indicating parasite clearance.

The most widely employed current method to monitor parasitological cure in clinical settings is PCR. However, PCR can be falsely negative when circulating parasite load is below the level of detection and simply hidden in the tissues. The only reliable readout is a positive PCR result which is a clear indicator for the presence of the parasite. Based on this information we considered two groups for the data analysis: i) Group “A” includes all PCR Pos patients whether they were treated or untreated (n = 514) because all of them had detectable parasites in blood; ii) Group “B” includes only treated PCR Neg individuals (n = 379) because of their higher likelihood having eliminated the parasite (Fig 2A). We excluded from the analysis the untreated PCR Neg patients (n = 761) as this subgroup likely contains most of the false negative PCR results (based on the rationale that they were not treated CC patients) (Fig 2A). Although group B introduces a bias into the analysis, we believe that based on the current state of the art to monitor parasite persistence/absence this option is the most agreeable to identify a parasite surrogate marker with this cohort.

Receiver Operating Characteristic (ROC) curves were calculated for the capacity of individual antigens to discriminate Group A from Group B. The Area Under the Curve (AUC) was used to define the antigens with the most discriminatory power. Supervised learning techniques (logistic regression, classification tree) were used to determine models to discriminate parasite persistence from parasite clearance. We preferred not to split the available data into training and validation data sets, but rather to perform cross validation, which provides an unbiased assessment of the model without reducing the training set. All statistical analyses were performed in SAS 9.4 (SAS Institute Inc., Cary, NC, USA).

## Results

### Selection of the most discriminant antigens

To test the hypothesis if an antibody biomarker(s) could serve as indicators of parasite persistence based on performance of PCR, we tested 1654 samples from the SaMi-Trop cohort using the MultiCruzi assay with 15 different antigen specificities (Fig 1A and 1B). The samples were derived from 455 patients with a reported history of BZN treatment and 1199 without any reported treatment. In 83% of the patients, the time between the reported treatment and study enrollment was more than five years. Among the 1199 untreated patients, 761 were PCR Neg (63.5%) and 438 PCR Pos (36.5%). The majority of the treated patients were PCR Neg (n = 379; 83.3%), with only 76 patients PCR Pos (16.7%). Fig 2A shows the breakdown with the relative proportion of each group within the 1654 tested samples.

First, we analyzed the results of the samples from both groups and recorded the discrete immunoreactivity of each individual antigen using a digital image analyzer. We next investigated the discriminatory power of each antigen separately using a logistic regression model based on the absolute intensity readings (using ROC curves as output). The analysis of the 15 ROC curves revealed Ag3 as the most discriminant antigen with an AUC of 0.74 for classification of patients into Group A or Group B (Table 1). When combining all 15 antigens in a logistic regression model, only a minor improvement in the classification resulted in an AUC of 0.79, illustrating the little additive contribution of the remaining antigens to the discriminative power of the model using Ag3 alone.

**Table 1:** Area under the curves (AUC) of each individual antigen showing separation power between the two analyzed groups A & B.

Test variables	AUC
Antigen 1	0.664
Antigen 2	0.622
Antigen 3	0.738
Antigen 4	0.698
Antigen 5	0.735
Antigen 6	0.653
Antigen 7	0.639
Antigen 8	0.623
Antigen 9	0.622
Antigen 10	0.672
Antigen 11	0.614
Antigen 12	0.465
Antigen 13	0.469
Antigen 14	0.617
Antigen 15	0.610

It is also interesting to note that Ag3 together with Ag2 reacted with the highest percentage of *T*. *cruzi* seropositive samples in untreated/treated patients and PCR positive/PCR negative individuals (Table 2). However, the difference of reactivity between untreated and treated groups is more important for Ag3.

**Table 2:** Reactivity of each antigen with seropositive samples according to different criteria (PCR and treatment). A sample is considered reactive on a given antigen if the score is superior to 0.

MultiCruzi Antigens	SaMi Trop cohort (n = 1654)			
	PCR Positive (%)	PCR Negative (%)	Treated Patients (%)	Untreated Patients (%)
1	94,55	91,23	88,79	93,58
2	99,42	99,12	98,90	99,33
3	99,22	97,46	96,70	98,50
4	98,25	94,30	89,67	97,75
5	96,69	91,75	85,49	96,25
6	97,08	96,14	95,38	96,83
7	80,54	78,60	68,57	83,24
8	69,84	64,82	60,88	68,47
9	63,04	60,70	52,09	64,97
10	93,39	90,96	87,03	93,49
11	80,16	77,28	66,81	82,49
12	8,37	8,60	7,69	8,84
13	28,99	35,26	29,89	34,61
14	95,53	91,93	92,09	93,41
15	79,18	75,44	73,85	77,65

### Ab3 as a surrogate marker to monitor parasite persistence

**Fig 3:**
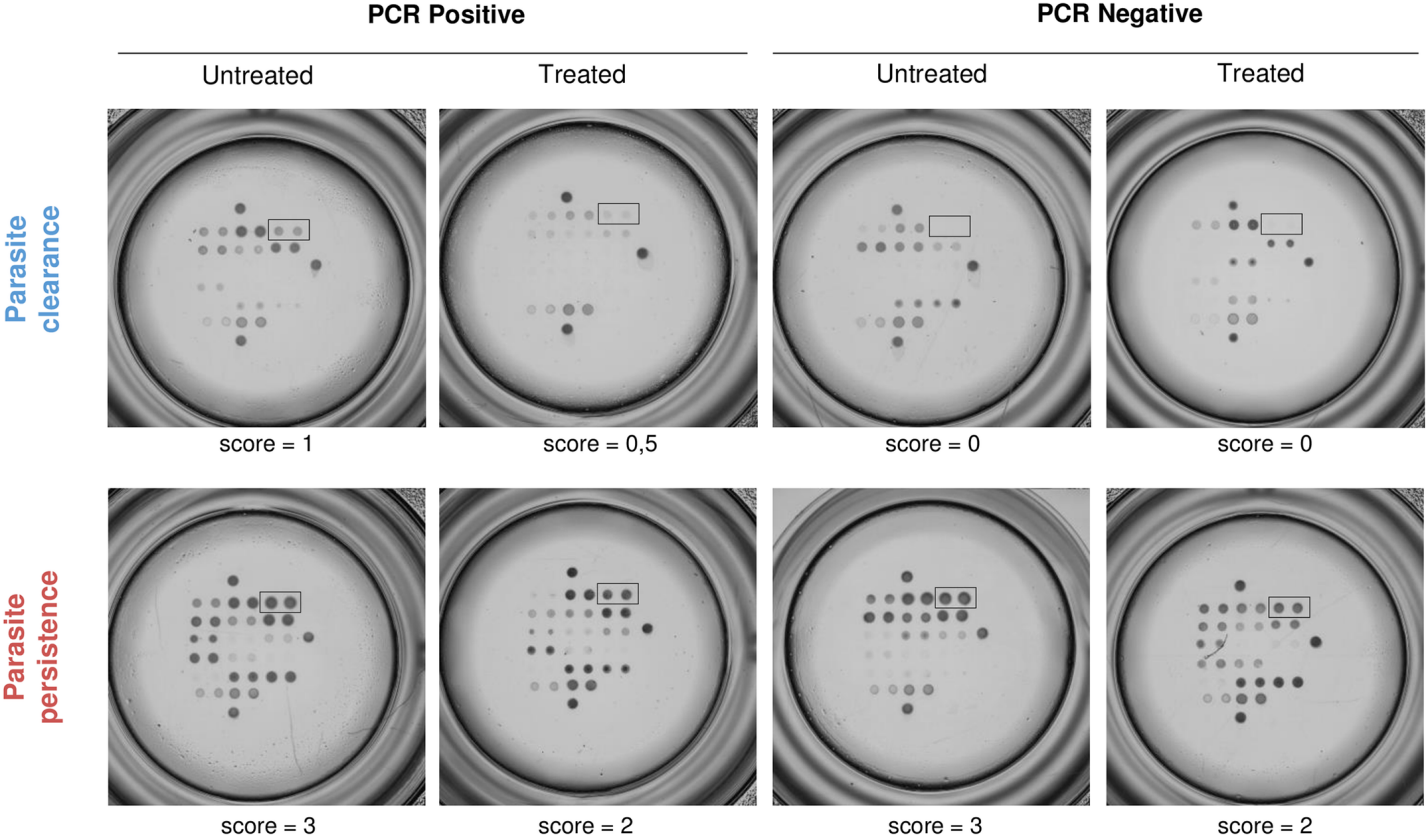
Serological profiles. Representative examples of serological profiles among each subgroup of the SaMi-Trop cohort. The black rectangle indicates Ag3. Ag3 reactivity’s in parasite persistence patients are high and low or absent in patients with cleared parasites.

In the present SaMi-Trop Cohort (n = 1654), Ab3 was reactive in 98% ((1654–33)/1654) of *T*. *cruzi* seropositive (PCR Neg and PCR Pos) samples as shown in Table 3. From this collection, 1362 samples showed high Ab3 reactivity scoring ≥ 2, and hence the samples were considered as likely from patients with parasite persistence (82.3%). The remaining samples (n = 292) with a score lower than 2 were classified as probable parasite clearance (17.7%). Table 3 depicts the overall distribution of Ab3 scores among each subgroup of the SaMi-Trop cohort and representative examples of Ab3 reactivities are illustrated in Fig 3.

**Table 3:** Distribution of Ab3 scores among each subgroup of the SaMi-Trop cohort.

Groups		Antibody 3 response (scores)					
		Parasite Clearance (proportions)			Parasite Persistence (proportions)		
		0	0.5	1	2	3	Total
**PCR Pos**	**Untreated**	4 (0,91%)	13 (2,97%)	21 (4,79%)	25 (5,71%)	375 (85,62%)	**438** **(100%)**
	**Treated**	0 (0%)	2 (2,63%)	3 (3,95%)	4 (5,26%)	67 (88,16%)	**76** **(100%)**
**PCR Neg**	**Untreated**	14* (1,84%)	32* (4,20%)	56* (7,36%)	68 (8,94%)	591 (77,66%)	**761** **(100%)**
	**Treated**	15 (3,96%)	36 (9,5%)	96 (25,33%)	38 (10,03%)	194 (51,19%)	**379** **(100%)**
**Total**		**33**	**83**	**176**	**135**	**1227**	**1654**

* Possible spontaneous parasite clearance or forgotten treatment.

**Fig 4:**
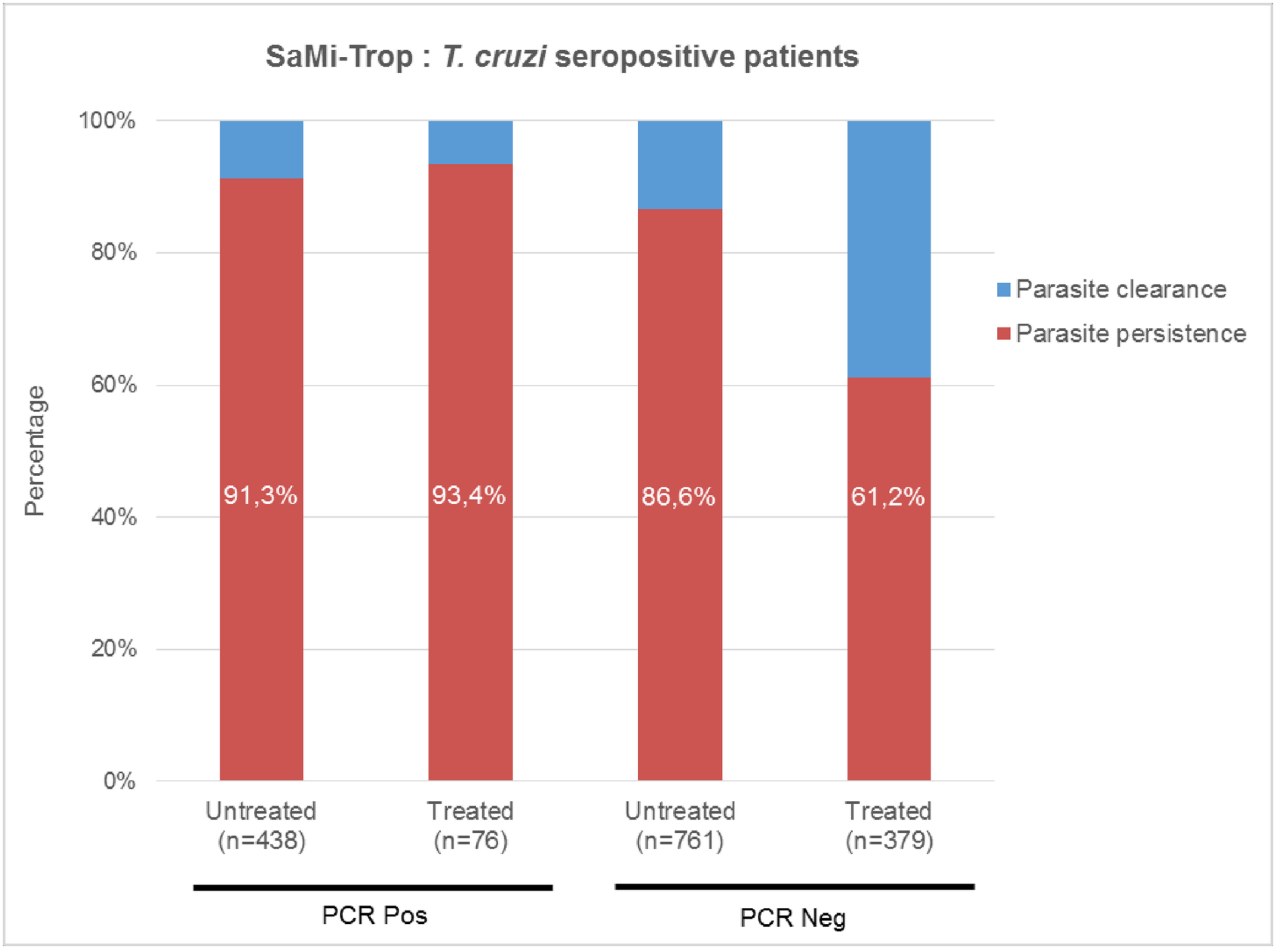
Ab3 is a surrogate marker to monitor parasite persistence. Distribution of subgroups parasite persistence and clearance in percentage in each subgroup of the SaMi-Trop cohort. When a score ≥ 2 is assigned to Ab3 serology, the patient is considered as having a persisting parasites (in red). If the Ab3 scores < 2 (signals below the cutoff) the patient has putatively cleared the parasite (in blue). Ab3 high scoring serology classifies about 90% of the patients to the parasite persistence group in both PCR Pos groups which contain circulating *T*. *cruzi* parasites.

Ab3 scores ≥ 2 were observed in 400 out of 438 (91.3%) of the untreated PCR Pos group patients, and in 71 out of 76 (93.4%) of the treated PCR Pos patients (Fig 4). In the untreated PCR Neg group, we observed a slightly lower rate of parasite persistence (659/761 = 86.6%) (Fig 4). Among the treated PCR Neg samples 61.2% (232/379) were classified as parasite persistence by the Ab3 serology (scores ≥ 2). Within this last subgroup there is a clear and significant shift towards lower signals of Ab3 (scores < 2) (147/379 = 38.8%) as compared to untreated PCR Neg patients (102/761 = 13.4%) (P < 0.0001). This difference may reflect the drug efficacy for clearing the parasite (Fig 4). A comparison between treated PCR Neg and treated PCR Pos groups with Ab3 scores ≥ 2 revealed a highly significant difference (P < 0.0001).

Remarkably, in the PCR Pos subgroups antibodies to Ag3 was measurable in 99.2% (510/514) of the patients. We only observed 4 patients with Ab3 score = 0 (absence of reactivity with Ag3), 15 patients with Ab3 score = 0.5 and 24 patients with Ab3 score = 1, while the vast majority (471/514 = 91.6%) had an Ab3 scores ≥ 2 (Table 3).

**Fig 5:**
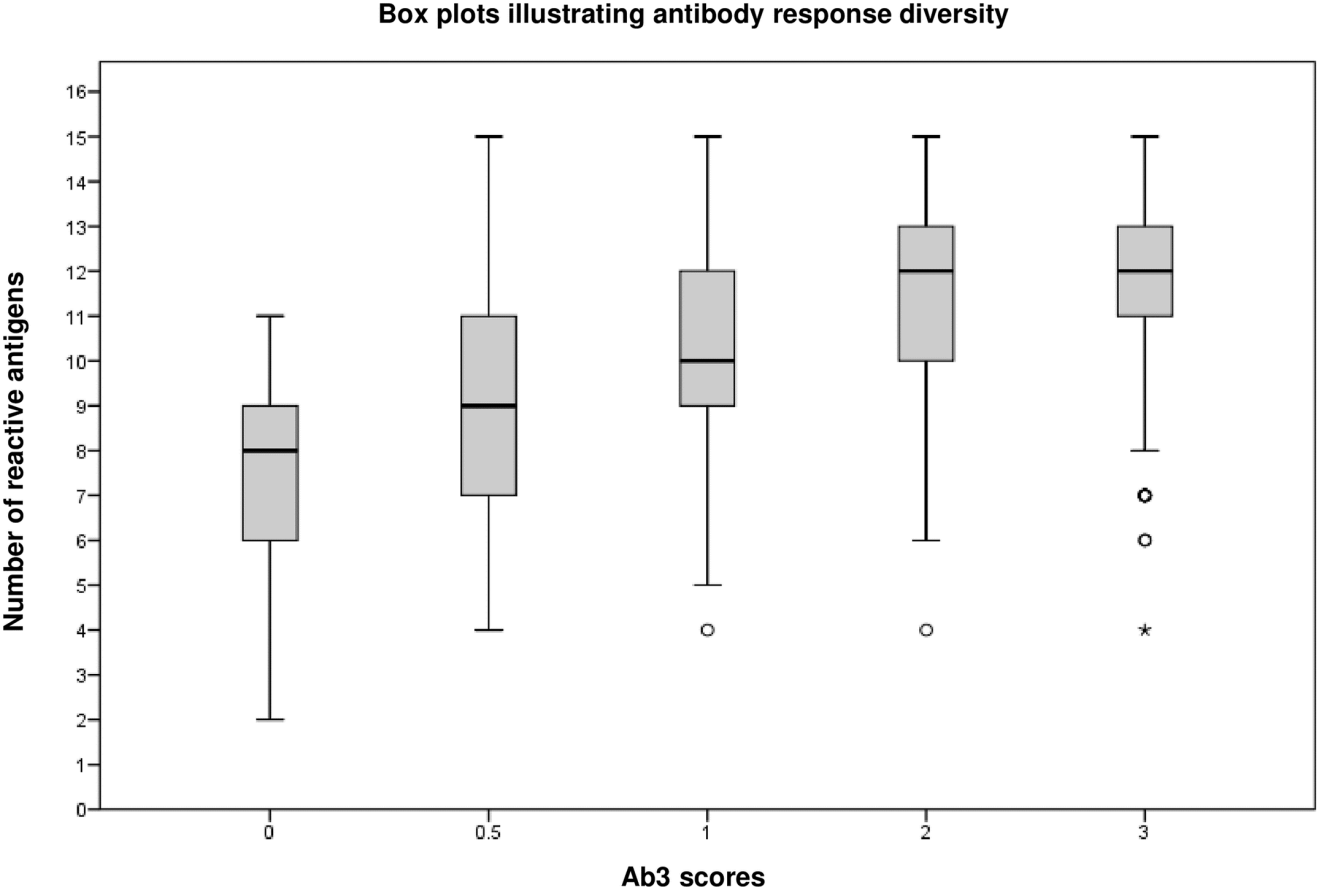
Box plots illustrating antibody response diversity. Box plots indicating the number of reactive antigens in function of Ab3 scores. We note that high scores (Ab3 ≥ 2) samples display large number of antigen reactivities as opposed to low scores (Ab3 < 2).

These data indicate that a high score on Ab3 seroreactivity *i)* strongly correlates with PCR Pos results (performance around 92%) and *ii)* ascertains a high number of parasite persistence cases within the PCR negative group, thus indicating that Ab3 serology is probably more informative than PCR. Thus high score Ab3 serology could be efficient for detecting parasite persistence in most *T*. *cruzi*-infected individuals. Ab3 score was also associated with total number of reactive antigens as shown on Fig 5. Suggesting an overall decline of the global immune response in samples with Ab3 scores <2.

### Establishment of the interpretation algorithm

Next, we sought to establish an interpretation algorithm to discriminate between group A and B. Using data input from all antigens, we applied different unsupervised learning techniques and we first performed a logistic regression analysis and, secondly a classification tree analysis to determine how appropriately the rules match with group A and B. Strikingly, all prediction models retained Ag3 as the most important antigen to discriminate group A from group B. To avoid overfitting, and to simplify the model, a final algorithm was proposed based on a threshold reactivity of Ab3 (reactivity scores of ≥ 2 or < 2). This threshold is obviously determined by the assay parameters and may be adjusted depending on new assay constraints. As shown in Table 3, the threshold of 2 for measuring Ab3 balances best the sensitivity for parasite persistence (PCR positive).

The prediction algorithm is intended to separate samples into one of the two likelihood groups: parasite clearance or parasite persistence, based on the measurement of a single specific antibody reactivity (Ab3). The interpretation algorithm is as follows: High-titers Ab3 are assessed with a score ≥ 2 (equivalent to high-signals in standard ELISA immunoassays). For a given patient, if Ab3 scores ≥ 2, the patient is considered as retaining parasite persistence; if the Ab3 scores < 2 the patient is considered to have cleared the parasite (Figs 2B and 1C). We retained this simple and robust algorithm to enable its ease-of-use and implementation in remote laboratories without need for sophisticated instrumentation such as digital readers and computed data analysis.

## Discussion

*T*. *cruzi-*infected patients demonstrate a large diversity in clinical forms as well as in the pattern of antibody responses, i.e., it is uncommon to find a single antigen that reacts with all seropositive sample [26] [29] [30]. Indeed, we previously described that a minimum of 4 antigens were required to confirm a sample as positive [26]. In this study we applied an innovative multi-parametric screening technology to identify antibody biomarker(s) that can be employed as surrogates to monitor parasite persistence. Building on an extensive epitope-mapping, we analyzed the diversity of antibody response against 15 selected *T*. *cruzi* antigens in a large sample collection from CC patients, either BZN-treated or untreated, and we further documented significant changes in these responses that were associated to therapy. Using an interdisciplinary approach, we explored our experimental data with different mathematical models and were able to show that one unique antibody (Ab3) is probably more sensitive than PCR to monitor for *T*. *cruzi* parasite persistence. Antigen 3 is derived from the microtubule associated proteins, we therefore, hypothesize that such protein is overexpressed during the replicative process of the parasite and thus stimulates the immune system for that particular specificity. Further experimental studies can be designed to consolidate this hypothesis.

This study touches one of the most important challenges in Chagas disease, which is the lack of reliable biomarkers to identify patients responding to drug treatment in a timely manner [31] [32] [33]. This is of special relevance because clinical symptoms are not useful indicators, due to the slowly progressive, and often irreversible, development of the disease and the lack of effective treatment particularly for the chronic phases of the disease. Several attempts have been made to identify biomarkers of therapeutic efficacy, including anti-*T*. *cruzi* antibodies (lytic antibodies, anti-Tc24, anti-F29), or more global host responses (anti-*T*. *cruzi* T-cell responses, transcriptomics and differential gene expression) [34] [35]. However, none of these biomarkers have led to a significant breakthrough [31]. Consequently, full sero-reversion, measured by conventional serology tests, currently helps to monitor parasitological cure, even though it is not suitable for patients living in endemic areas nor for conducting clinical trials, since it requires multiyear follow-up. Indeed, sero-reversion measured by conventional serology takes up to 20 years [7]. Because screening assays are typically designed and optimized to pick up low level antibodies indicative of past infections, they cannot be instrumental for monitoring purposes. PCR techniques, used in clinical trials, allow rapid and accurate recognition of treatment failure but cannot reliably confirm parasite clearance [32] [36]. Other limitations of PCR to monitor therapeutic outcomes include difficulty to perform molecular assays in resource-poor settings (special lab infrastructure and well-trained technologists), cost, and the requirement for at least 10ml of patient blood that is processed quickly after phlebotomy.

Our findings suggest that a single specific antibody could be a valuable biomarker to detect and track an active infection in most *T*. *cruzi*-infected individuals. This conclusion is based on the observations that Ab3: i) is induced at high levels in *T*. *cruzi* seropositive sera, ii) has superior sensitivity for parasite persistence/clearance as compared to PCR, indicating that PCR may overestimate the trypanocidal effects of drugs and iii) outperforms standard serology to monitor parasite clearance in the tested CC population. Regarding the last point, it is interesting to note that Sánchez Negrette et al. [37] have shown that a discrete analysis of a limited number of antigens classically integrated in diagnostic assays, have faster regression trends in response to treatment in comparison with the global ELISA signals collectively generated by large number of antigens.

Importantly, our findings observed with Ab3 serology reflect efficacy rates in chronic adult *T*. *cruzi*-infected patients. Depending on the methodology (serology, respectively molecular or parasitological methods), geographical regions and the time elapsed after treatment evaluation, reported drug efficacy rates are highly variable when drugs are administered during the chronic phase of infection [31] [38]. When complete sero-reversion is used as a gold standard, rates vary approximately between 10% and 45%; Viotti suggested 8% and Fabbro 40% efficacy [11] [13]. The large randomized BENEFIT trial with benznidazole, reported 66% of parasite clearance (PCR Neg) at end of treatment among those with PCR positive results at baseline. This proportion is reduced to 46.7% after 5 years which highlights under performance of PCR techniques for hidden parasites [14]. Striking regional differences were shown, with PCR conversion rates higher in Argentina, Bolivia and Brazil than in Colombia and El Salvador.

To calculate the presumably efficacy rate of benznidazole in the selected population of the SaMi-Trop cohort (Brazilian CC patients) we focused on the subgroup of treated-PCR Neg with Ab3 scores < 2 because treated patients that remained PCR Pos convey a treatment failure message. In the treated-PCR Neg CC patients, Ab3 serology identified significantly higher parasite clearance rate (147/379 = 38.8%) as compared to the untreated PCR Neg individuals (102/761 = 13.4%). If we assume Ab3 classification is correct, then the parasite clearance attributable to the BZN-treatment can be estimated at 25.4% (38.8%-13.4%) (Table 3). A small proportion in the untreated- and treated-PCR Neg groups may also result from spontaneous parasite elimination (Table 3). Spontaneous cure, in the absence of treatment, has been documented in long-term untreated *T*. *cruzi*-infected subjects, however, the mechanisms underlying this phenomenon have not yet been explored nor reliably documented. The results described in this study indicate that the applied interdisciplinary approach, using experimental data from the discrete analysis of well-selected antigens combined with mathematical modeling, can be an excellent strategy to identify novel surrogate marker(s).

Main limitations in this study are that we did not have the samples prior to the treatment, and BZN treatment information was obtained through a questionnaire. To further validate the data we now aim at analyzing Ab3 serology across various *T*. *cruzi*-infected populations and cohorts. Studies testing longitudinal samples, characterized by PCR, from treated patients monitored over several years pre/post BZN treatment are in progress to define if quantitative Ab3 serology can indeed be a reliable and early sign of treatment efficacy or even spontaneous cleansing of the parasitic load.

These studies should establish if Ab3 could become a suitable tool to obtain parasitological endpoints, complementing and possibly obviating the need for PCR, to support the development of improved drugs, earlier treatment and better clinical management of Chagas disease patients. Indeed, the serological detection of Ab3 has the potential to both act as a treatment efficacy indicator and could also serve as a flag which guides the medical decision towards therapy.

## Supporting information

S1 Appendix Sample flow chart from the collection of SaMi-Trop study cohort through MultiCruzi testing. (DOCX)

S2 Appendix STARD 2015 checklist. (DOCX)
