# Supplementary material for: A novel antibody surrogate biomarker to monitor parasite persistence in Trypanosoma cruzi-infected patients
Source: PLoS Negl Trop Dis. 2018 Feb 9;12(2):e0006226. doi: 10.1371/journal.pntd.0006226 (PMC5823467; doi:10.1371/journal.pntd.0006226)
Supplement: S1 Appendix — (DOCX) [file pntd.0006226.s001.docx]

**Support Information Files**

S1: Sample flow chart from the collection of SaMi-Trop study cohort through MultiCruzi testing.

Note:

No reference standard tests available for the assessment. Detailed data are presented in Table 3 of the manuscript.
