# Supplementary material for: A novel antibody surrogate biomarker to monitor parasite persistence in Trypanosoma cruzi-infected patients
Source: PLoS Negl Trop Dis. 2018 Feb 9;12(2):e0006226. doi: 10.1371/journal.pntd.0006226 (PMC5823467; doi:10.1371/journal.pntd.0006226)
Supplement: S2 Appendix — (DOCX) [file pntd.0006226.s002.docx]

| **Section & Topic** | **No** | **Item** | **Reported on page #** |
| --- | --- | --- | --- |
| **TITLE OR ABSTRACT** |  | **A Novel Antibody Surrogate Biomarker to Monitor Parasite Persistence in *Trypanosoma cruzi-*Infected Patients** | **1** |
|  | **1** | Identification as a study of diagnostic accuracy using at least one measure of accuracy  (such as sensitivity, specificity, predictive values, or AUC) |  |
| **ABSTRACT** |  |  |  |
|  | **2** | Structured summary of study design, methods, results, and conclusions  (for specific guidance, see STARD for Abstracts) | 2 |
| **INTRODUCTION** |  |  |  |
|  | **3** | Scientific and clinical background, including the intended use and clinical role of the index test | 4, 5 |
|  | **4** | Study objectives and hypotheses | 6 |
| **METHODS** |  |  |  |
| *Study design* | **5** | Whether data collection was planned before the index test and reference standard  were performed (prospective study) or after (retrospective study) | 6 |
| *Participants* | **6** | Eligibility criteria | 6 |
|  | **7** | On what basis potentially eligible participants were identified  (such as symptoms, results from previous tests, inclusion in registry) | 6 |
|  | **8** | Where and when potentially eligible participants were identified (setting, location and dates) | 6 |
|  | **9** | Whether participants formed a consecutive, random or convenience series |  |
| *Test methods* | **10a** | Index test, in sufficient detail to allow replication | 7 |
|  | **10b** | Reference standard, in sufficient detail to allow replication | 7, 8 |
|  | **11** | Rationale for choosing the reference standard (if alternatives exist) |  |
|  | **12a** | Definition of and rationale for test positivity cut-offs or result categories  of the index test, distinguishing pre-specified from exploratory | 8 |
|  | **12b** | Definition of and rationale for test positivity cut-offs or result categories  of the reference standard, distinguishing pre-specified from exploratory |  |
|  | **13a** | Whether clinical information and reference standard results were available  to the performers/readers of the index test | 8 |
|  | **13b** | Whether clinical information and index test results were available  to the assessors of the reference standard |  |
| *Analysis* | **14** | Methods for estimating or comparing measures of diagnostic accuracy |  |
|  | **15** | How indeterminate index test or reference standard results were handled |  |
|  | **16** | How missing data on the index test and reference standard were handled | 6, 8 |
|  | **17** | Any analyses of variability in diagnostic accuracy, distinguishing pre-specified from exploratory |  |
|  | **18** | Intended sample size and how it was determined |  |
| **RESULTS** |  |  |  |
| *Participants* | **19** | Flow of participants, using a diagram |  |
|  | **20** | Baseline demographic and clinical characteristics of participants | 6 |
|  | **21a** | Distribution of severity of disease in those with the target condition |  |
|  | **21b** | Distribution of alternative diagnoses in those without the target condition |  |
|  | **22** | Time interval and any clinical interventions between index test and reference standard |  |
| *Test results* | **23** | Cross tabulation of the index test results (or their distribution)  by the results of the reference standard | 17 |
|  | **24** | Estimates of diagnostic accuracy and their precision (such as 95% confidence intervals) |  |
|  | **25** | Any adverse events from performing the index test or the reference standard |  |
| **DISCUSSION** |  |  |  |
|  | **26** | Study limitations, including sources of potential bias, statistical uncertainty, and generalisability | 11, 12 |
|  | **27** | Implications for practice, including the intended use and clinical role of the index test | 13 |
| **OTHER INFORMATION** |  |  |  |
|  | **28** | Registration number and name of registry |  |
|  | **29** | Where the full study protocol can be accessed |  |
|  | **30** | Sources of funding and other support; role of funders |  |

S2: STARD 2015 checklist
